# Role of extracellular RNA-carrying vesicles in cell differentiation and reprogramming

**DOI:** 10.1186/s13287-015-0150-x

**Published:** 2015-09-03

**Authors:** Peter J. Quesenberry, Jason Aliotta, Maria Chiara Deregibus, Giovanni Camussi

**Affiliations:** Department of Medicine, Warren Alpert Medical School of Brown University, Box G-A1, Providence, RI 02912 USA; Translational Center for Regenerative Medicine, University of Torino/Fresenius Medical Care, via Nizza 52, 10126 Torino, Italy; Department of Medical Sciences, University of Torino, Corso Dogliotti 14, 10126 Torino, Italy

## Abstract

Growing evidence suggests that transcriptional regulators and secreted RNA molecules encapsulated within membrane vesicles modify the phenotype of target cells. Membrane vesicles, actively released by cells, represent a mechanism of intercellular communication that is conserved evolutionarily and involves the transfer of molecules able to induce epigenetic changes in recipient cells. Extracellular vesicles, which include exosomes and microvesicles, carry proteins, bioactive lipids, and nucleic acids, which are protected from enzyme degradation. These vesicles can transfer signals capable of altering cell function and/or reprogramming targeted cells. In the present review we focus on the extracellular vesicle-induced epigenetic changes in recipient cells that may lead to phenotypic and functional modifications. The relevance of these phenomena in stem cell biology and tissue repair is discussed.

## Introduction

**Fig. 1:**
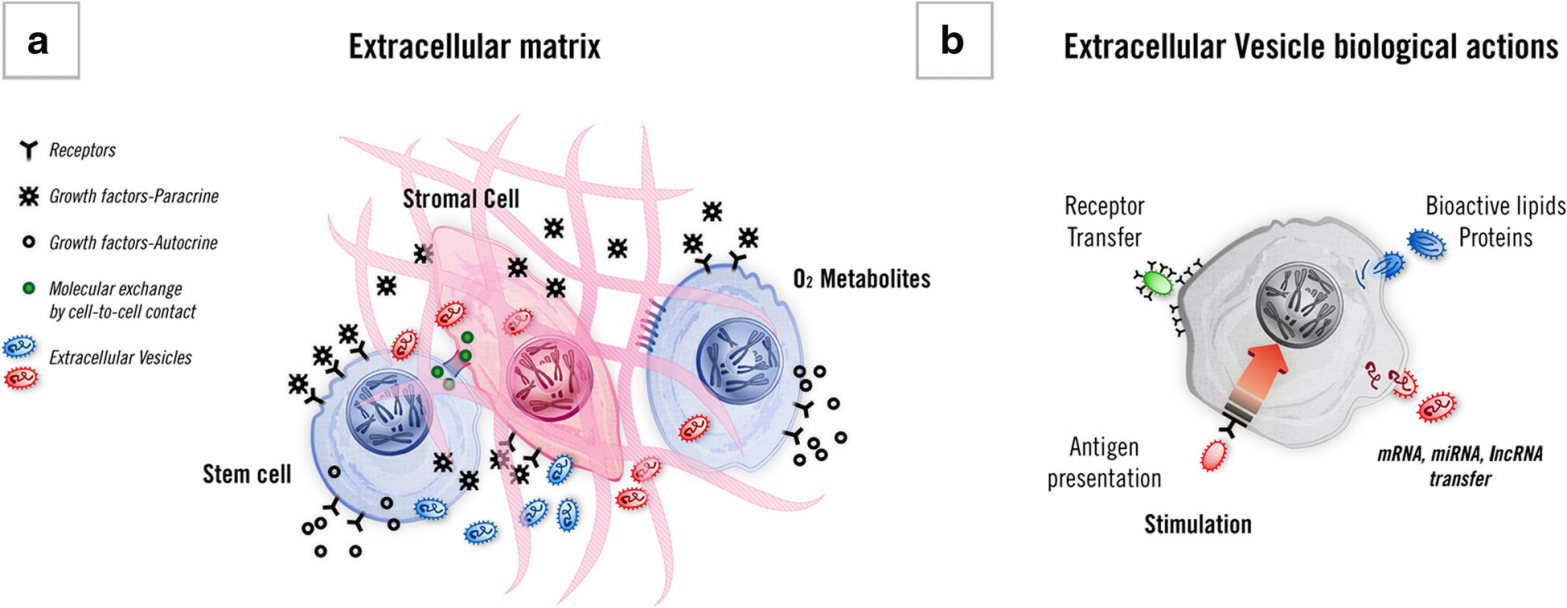
Combined factors that modulate cell fate and functions. **a** Soluble growth factors may act as paracrine or autocrine mechanisms by interacting with cell receptors directly or after binding to matrix; extracellular matrix and direct cell-to-cell contact may in turn direct cell fate in a defined microenvironment. The interaction between stem and stromal cells is reciprocal. In addition, oxygen tension and metabolic products may modulate cell phenotype. Extracellular vesicles are part of this complex regulatory network of factors involved in the interaction between cells. **b** Schematic representation of different modes of action of extracellular vesicles. *lncRNA* long noncoding RNA, *miRNA* microRNA

Information exchange between cells coordinates development and functional interplay in complex organisms. Cells can communicate via physical interactions, including membrane bridge formation, such as tunneling nanotubes and cytonemes, and/or through the release of soluble factors [1–3]. The fate of the cell is determined by coordinated and dynamic interactions among a number of factors, acting in a defined microenvironment. In particular, stem cells are highly sensitive to extracellular signals that play a critical role in maintenance of stem cell characteristics, differentiation, and interplay with somatic cells. A tight spatial and timing regulation of growth factor action during embryonic development has been suggested [4]. Growth factors may act either in an autocrine or a paracrine fashion and their temporal and spatial concentration modulates the cell phenotype and function. In this context, extracellular matrix also has a critical role because it may limit, in a defined niche, the action of growth factors since it often binds growth factors and may deliver cell fate-determining signals by direct interaction with cells [5, 6]. Several other environmental factors including oxygen concentration and mechanical, metabolic, and biochemical conditions have been shown relevant in cell differentiation and have been reviewed extensively (Fig. 1) [3]. Similarly, reprogramming of somatic cells involves a complex interaction among intracellular and extracellular signals leading to epigenetic remodeling [6]. The cell phenotype is therefore determined by signals that target the cells received within a defined microenvironment. This process involves the ability of cells to change phenotype depending upon specific signals.

Cell-secreted vesicles have emerged as an integral component of intercellular exchange of information (Fig. 1). This concept is based on the observation that vesicles may transfer different types of signals between cells [7, 8].

Classification of vesicles into exosomes, originating from the membrane of the endosomal compartment, and microvesicles, derived from plasma membrane budding, is based on their biogenesis [9]. However, given the overlapping features of exosomes and microvesicles, and the variability of content and biogenesis depending on cellular type, the term extracellular vesicles (EVs) has been suggested to include the different types of vesicles [10].

During vesiculation, bioactive lipids and receptors remain associated with vesicle membranes, and cytosolic proteins and nucleic acids are contained within the vesicles [11]. Surface-expressed lipids and receptors derived from donor cells may allow interaction and membrane fusion or internalization of vesicles within recipient cells and may lead to cell activation.

## Biological activities of extracellular vesicles

Several studies have emphasized the role of the bioactive lipid and protein content of EVs in their function [7–9, 11, 12]. EVs may act as a signaling complex or by delivering proteins, bioactive lipids, or receptors leading to activation of target cells (Fig. 1b). Early studies by Raposo et al. [13] showed that B lymphocyte-derived vesicles induced an antigen-specific major histocompatibility restricted T-cell response. Based on the presence of vesicles on the surface of antigen presenting cells, it has been suggested that they may act as a vehicle for major histocompatibility class II–peptide complex. Subsequent studies further supported the concept that antigen presenting cells may exploit vesicles for antigen presentation [14]. The acquisition of receptors by bystander B cells has also been shown to depend on the transfer of membrane from activated B cells allowing an expansion of the antigen-binding B cells [15]. This was confirmed for several other receptors, including the transfer of the adhesion molecules from platelets to tumor [16] or endothelial cells [17] resulting in enhanced proadhesive properties. Moreover, the EV-mediated transfer of Fas ligand from tumor cells to activated T cells has been shown to induce T-cell apoptosis leading to tumor immune escape [18].

In addition, EVs were shown to be a vehicle for the exchange of bioactive lipids, proteins, and receptors between cells that, in the context of the tumor microenvironment, could change the stromal cell phenotype and favor tumor invasion and metastasis [19]. The role of EV-transported bioactive lipids is currently undervalued. However, angiogenic activity of sphingomyelin present on the surface of EVs released by cancer cells has been reported and shown to account for the enhanced endothelial cell migration and invasion [20]. Conversely, a large body of information is available regarding the exchange of proteins and receptors by means of EVs. For example, it has been shown that the EV-mediated transfer of membrane-bound CX3CL1/fractalkine enhanced cell invasiveness [21]. In addition, cancer cell-derived EVs may enhance tumor invasion by supplying matrix metalloproteinases [21]. Similarly, the EV-mediated transfer of tissue transglutaminase and fibronectin from breast carcinoma and glioma cells was shown to transform fibroblasts and epithelial cells [22]. Moreover, remodeling of tissue matrices and activation of endothelial cells at distant sites by tumor-derived EVs may favor the formation of the “premetastatic niche” [23–25].

Several studies have indicated that tumor-derived EVs may facilitate immune escape. Indeed, EVs released by prostate cancer cells express the Fas ligand and may induce cytotoxic T-lymphocyte apoptosis [18]. Furthermore, EVs released by renal cancer stem cells were found to be enriched in fibroblast growth factor, vascular endothelial growth factor, ephrin A3, angiopoietin 1 and matrix metalloproteinase 2/9, which may promote angiogenesis and formation of a premetastatic niche in the lung [25].

More recently, it has been suggested that membrane vesicles may act as transcription modulators and influence cell phenotypes [26]. This evolutionarily conserved mechanism allows exchange of genetic information between cells, as vesicles encapsulate and protect DNA, mRNA, long noncoding RNA (lncRNA) and microRNA (miRNA) from degrading enzymes [27–29]. EVs may serve to specifically target extracellular RNA (exRNA) to cells expressing counter-receptors, thus allowing vesicle uptake from recipient cells [30]. Encapsulated RNA is not the only form of enzyme-protected exRNA [31, 32] present in the biological fluids, because RNA binding proteins, such as proteins of the argonaute family and high-density and low-density lipoproteins, may also confer nuclease resistance. Recent studies have shown a critical role of RNA-binding proteins in pluripotency, stem cell differentiation, and cell reprogramming (for review see [33]). Moreover, it has been shown that miRNAs modulate the extracellular matrix and play a critical role in regulation of somatic cell reprogramming [34].

Taken together, these studies indicate that EVs, owing to their complex composition, may deliver different signals to the recipient cells which may modify cell function and phenotype. Conceivably, different bioactive molecules may synergize in the EV biological actions.

## Extracellular vesicles as vehicles for transfer of genetic information

Several studies have demonstrated that encapsulated mRNA can be shared between cells. The horizontal transfer of vesicle-encapsulated mRNA was shown to reprogram hematopoietic progenitors [35] and quiescent endothelial cells [36]. Ratajczak et al. [35] demonstrated that microvesicles obtained from murine embryonic stem cells improved survival and expansion of lineage-negative Sca-1-positive progenitors by enhancing the expression of Nanog, Oct-4, and Rex-1 and of HoxB4, Scl, and GATA 2, which are markers of early pluripotent stem cells and of hematopoietic stem cells, respectively. These phenotypic changes were paralleled by mitogen-activated protein kinase p42/44 and serine-threonine kinase AKT phosphorylation. The mRNA coding for several pluripotent transcription factors enriched within microvesicles was transferred and translated into proteins, and RNA inactivation was found to inhibit the biologic activity of these microvesicles, suggesting a relevant role for vesicle-shuttled mRNA. Transferred mRNA may thus trigger epigenetic changes in the recipient cells. This implies translation of mRNA into proteins, as also shown by Valadi et al. [37] in mouse and human mast cells; their study showed that mast cell-derived exosomes contain mRNA from about 1300 different genes, which was proven to be functional because it could be translated into protein in vitro. The transient production of green fluorescent protein (GFP) by cells that have incorporated vesicles containing GFP mRNA further supports the delivery of functional mRNA [38]. Furthermore, we also observed in-vivo translation of mRNA in mice treated with vesicles derived from human mesenchymal stem cells [38, 39]. Aliotta et al. [40] demonstrated that the delivery of mRNA by EVs, as well as the induction of transcription, can account for the expression of tissue-specific RNA in bone marrow cells. The vesicle-mediated transfer of lung mRNA to bone marrow cells induced the expression of Clara cell-specific protein, surfactant A–D, and aquaporin-5 mRNAs and protein in the recipient cells. Bone marrow cells were thus shown to have acquired a lung phenotype.

Recently, Ridder et al. [41] showed an EV-mediated transfer of Cre mRNA used as a reporter gene from blood cells to neurons. The observation of an intercellular transfer of functional mRNA reveals that, in inflammatory conditions, hematopoietic cells may communicate with different organs, including the brain. EVs released by embryonic stem cells may also transfer embryonic stem cell mRNAs, such as for Oct4 and Sox2, implicated in the preservation of pluripotency, to retinal progenitor Muller cells, along with mRNAs related to embryonic and early retinal genes [42]. Human milk-derived EVs carry mRNA transcripts and reverse transcriptase, and may transfer genetic information from the mother to the neonate. Reverse transcription and integration into the genome of transcripts carried by EVs from a healthy wet nurse have also been suggested to correct clinical expression of genetic diseases [43].

A fraction of mRNA present in exosomes has been reported recently to be characterized by a specific pattern of fragmentation with the presence of 3′ ends containing elements that, being rich in miRNA-binding sites, may compete with the mRNAs of recipient cells, thus modulating their translation [44]. Molecules carried by EVs that can modify the cell phenotype include miRNAs known to control genes encoding most proteins [28] and lncRNAs known to modulate the epigenome [45].

The presence of miRNAs within exosomes released from mast cells and their transmission from one cell to another was shown by Valadi et al. [37]. An enrichment of miRNAs was also detected in vesicles derived from mouse embryonic fibroblasts [46]. Chen et al. [47] showed that 55–65 nm “microparticles” secreted by human embryonic mesenchymal stem cells are enriched in pre-miRNAs. These small RNAs, not associated with the Argonaute 2 (Ago2) protein, were suggested to be encapsulated in cholesterol-rich vesicles since they are sensitive to RNase after phospholipase A2 and detergent treatment. In-vitro treatment with RNase III was shown to generate mature miRNAs suggesting that, once incorporated into cardiomyocytes, pre-miRNAs may be processed to miRNAs [47]. We found that EVs released from human adult bone marrow-derived mesenchymal stromal cells (MSCs) contain mature miRNAs and that miRNAs encapsulated in vesicles were more abundant than in the cell of origin, suggesting a specific compartmentalization [48]. Gibbings et al. [49] suggested that packaging of RNA into monocyte-derived exosomes may occur within multivesicular bodies following an interaction with components of miRNA effector complexes, such as Ago2 and GW182.

Studies on the comparison of miRNA families present in vesicles and in the originating cells, as well as ribonucleoproteins implicated in RNA intracellular handling, have provided additional information on miRNA compartmentalization. We have found that several stress granule-specific proteins are present within stem cell-derived vesicles [48]. These proteins include ribonucleoproteins involved in the storage of RNA such as Stau 1 and 2, TIA, TIAR, and HuR, known to be expressed in the nucleus and in stress granules but absent in processing bodies. Moreover, adult human MSC-derived vesicles have been shown to contain Ago2 [48], which is involved in the transport and maturation of miRNAs. Laffont et al. [50] demonstrated that platelet-derived EVs carry functional Ago2–miRNA complexes able to regulate gene expression in the endothelial cells. Moreover, it was found that the presence in cell-secreted EVs of Ago2 complexes is critical for miRNA stability [51] and function [52]. The selective export of miRNAs in EVs has also been linked to Ago2 in multiple cell types, suggesting a common mechanism for loading of miRNA in EVs [53].

Experiments based on chemical inhibition or on knockdown of neutral nSMase2, an enzyme involved in the synthesis of ceramide, uncovered the role of lipids in miRNA compartmentalization within exosomes [54, 55]; inhibition of neutral nSMase2 activity resulted in reduced exosome content of miR-16 and miR-146a.

EV treatment influences the translation of protein targets of specific miRNAs, so we can deduce that EV-delivered miRNAs must be functional [48]. Many studies in the literature have demonstrated that miRNAs can be transported by EVs to other cells. For instance, the tumor-suppressive miR-143 has been shown to be transferred from normal prostate cells to cancer cells by means of exosomes, inducing suppression of its target genes and preventing cancer cell growth [56]. In addition, Epstein–Barr virus (EBV)-infected cells can secrete exosomes containing mature EBV-encoded miRNAs that silence B-cell genes, causing persistence of infection [57]. EVs released from monocytes/macrophages in culture contain miR-150 and are able to transfer this miRNA to endothelial cells, inducing downregulation of c-Myb and enhancing migration [58].

Changes in gene expression induced in retinal Muller cells by EVs from embryonic stem cells have also been ascribed to miRNA transfer [42]. Enhanced expression of miRNA that regulates early ocular genes and genes relevant for retina remodeling and protection, and the activation of a de-differentiation and pluripotency program were observed. On the other hand, downregulation of miRNAs involved in cell differentiation and in inhibition of cell proliferation has been shown to be triggered by embryonic stem cell EVs [42].

EV-mediated transfer of miRNAs has also been implicated in the immune synapsis between T cells and antigen presenting cells [59]. In addition, miRNA-carrying EVs have been suggested to allow communication between dendritic cells, amplifying their function [60].

Some studies have reported that EVs may also contain DNA. EVs derived from mouse cardiomyocytes were shown to contain 343 chromosomal DNA sequences that can be translocated to the cytosol and nuclei of target fibroblasts [61]. EV-mediated transfer of DNA may concur with the phenotypic changes that occur in cardiac remodeling after injury. The presence of mitochondrial DNA has also been identified in EVs released from cancer cells [62].

EVs produced by tumor cells were also shown to deliver retro-transposon elements and amplified oncogene sequences to endothelial and stromal cells [63], thus inducing changes in the microenvironment that promoted tumor growth and progression. Another important finding is the transfer of the human H-ras oncogene to nontransformed cells through EVs released by cancer cells [64]. Al-Nedawi et al. [65] demonstrated that EVs released by human glioma cells may account for horizontal propagation of oncogenes, leading to phenotype changes in different subsets of tumor cells.

## The role of cellular phenotype changes induced by extracellular vesicles in stem cell biology

The pivotal study by Ratajczak et al. [35] showed that vesicle-mediated signaling was critical for the preservation of stemness and pluripotency of hematopoietic stem/progenitor cells, and was attributed to the delivery of proteins and mRNA.

There is still an ongoing debate about stem cell plasticity [66]. The Quesenberry group studied the plasticity of stem cells with regard to the ability of bone marrow cells to acquire the phenotype of nonhematopoietic cells, in particular regarding the lung [67–69]. After injection of bone marrow cells expressing GFP into lethally irradiated mice, GFP-positive pulmonary epithelial cells were detected in the lungs [70]. Co-culture experiments of murine bone marrow cells with lung tissue separated by a cell-impermeable membrane showed that bone marrow cells were subsequently expressing specific mRNA of lung cells, such as Clara cell-specific protein, aquaporin-5, and surfactants A–D [71]. Expression levels of this mRNA were significantly enhanced when injured lungs obtained from irradiated mice were used. Studies performed on conditioned media by differential ultracentrifugation demonstrated that a mixture of microvesicles and exosomes had greater activity then exosomes alone.

To investigate the mechanisms underlining lung mRNA induction in bone marrow cells after exposure to EVs, co-culture experiments in trans-wells of rat lung with bone marrow from mice or of mouse lung with bone marrow from rats were performed [72]. By using species-specific primers for surfactants B and C, an immediate increase in mRNA of both mouse and rat origin was found. When cells were kept in liquid culture supplemented with cytokines, the mouse bone marrow cells previously co-cultured with rat lung rapidly terminated the expression of rat mRNA surfactant, but maintained the expression of mouse mRNA for up to 12 weeks [72].

These results suggest a rapid transfer of rat mRNA to bone marrow cells with its subsequent degradation, and the transfer of transcription factors able to trigger the expression of murine mRNA for surfactants B and C. This phenomenon was partially sensitive to RNase treatment, so the persistency of epigenetic changes observed in bone marrow cells was interpreted as being due to the transfer of noncoding regulatory RNAs, such as miRNAs and lncRNAs [72] (Fig. 1). Using PKH26-labeled and CSFE-labeled EVs, the biological activity of EVs was found to be related to their entry into the cells [40] as well as being related to the expression of adhesion molecules on the EV surface [73]. Co-culture in trans-wells of murine bone marrow cells with other organs such as the heart, liver, and brain also induced the expression of tissue-specific mRNAs [40].

These experiments indicate that vesicles derived from various organs may induce phenotypic changes in bone marrow cells, shedding new light on stem cell plasticity. EV-mediated exchange of genetic information has therefore been suggested as a fundamental component of the continuum model of stem cell biology, proposed by Quesenberry and colleagues, where transit into the cell cycle and the environmental stimuli are critical for the differentiation decision of stem cells [74].

## The role of stem cell-derived extracellular vesicle-mediated cell fate alterations in tissue injury repair

In the context of tissue injury, EV-mediated exchange of information could be bidirectional between stem and injured cells.

Repair of acute kidney injury (AKI) induced by MSCs is a good model to study tissue regeneration in the absence of stem cell contribution due to stem cell plasticity. Administration of MSCs was found to induce AKI recovery. However, unlike hematopoietic stem cells which are able to engraft in the kidney [75], MSCs only transiently localize in the injured kidney without permanent engraftment. Humphreys et al. [76] showed that MSC-induced AKI recovery must be ascribed to an intrinsic capacity of epithelial cells to repopulate the injured tubules. Using a genetic fate-mapping technique, these authors demonstrated that the predominant mechanism of renal tubule repair after ischemic injury is the re-entry of surviving tubular cells into the cell cycle, with consequent proliferation due to mesenchymal de-differentiation. This process has been named “epithelial–mesenchymal–epithelial cycling” [77]*.* A paracrine/endocrine action of MSCs has been suggested by the experiments of Bi et al. [78], who showed that the effect of MSCs was reproduced by their conditioned medium, which diminished apoptosis, enhanced survival, and reduced injury in cisplatin-induced AKI. The involvement of a paracrine/endocrine mechanism in the regenerative properties of MSCs for the recovery of other organs, such as the liver or heart, has also been described [79].

**Fig. 2:**
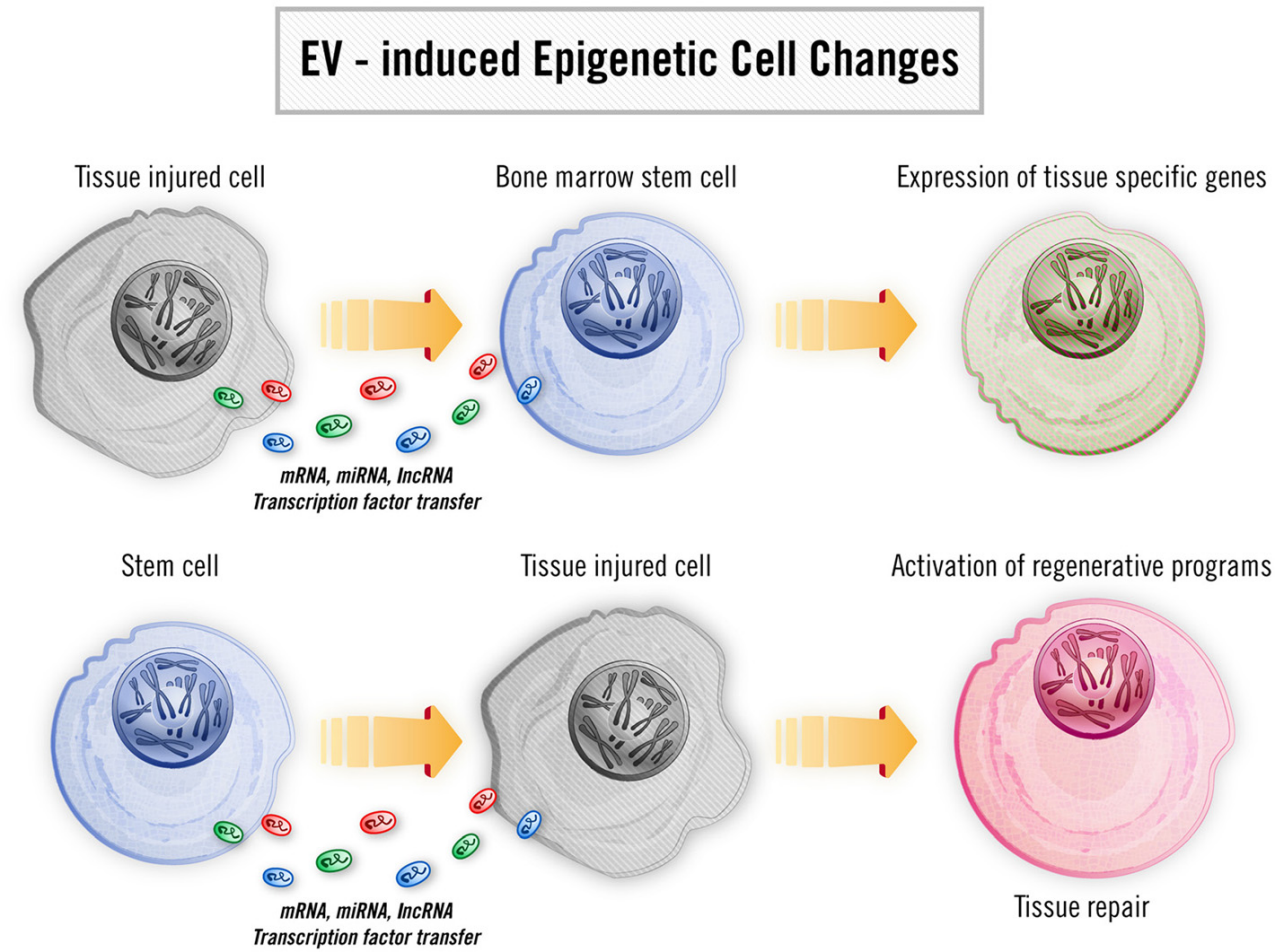
Model of extracellular vesicle-induced modulation of cell phenotype involved in the repair of tissue injury. *EV* extracellular vesicle, *lncRNA* long noncoding RNA, *miRNA* microRNA

The modulation of cell fate by EVs has been implicated in MSC paracrine/endocrine action. We compared the effect of MSC-derived EVs with that of the cell of origin in an experimental model of AKI induced in SCID mice by intramuscle injection of glycerol [38]. EVs were found to be able to mimic the effect of MSCs by promoting tubular cell proliferation and resistance to apoptotic injury, leading to functional and morphological recovery of AKI. The major role of RNA transfer in the biological action of EVs was demonstrated by experiments involving inactivation of RNA. In addition, the translation to protein of human MSC-specific mRNA was observed both in vitro and in vivo in murine tubular epithelial cells [38] (Fig. 2). Experiments showing the transfer of human insulin-like growth factor 1 (IGF-1) receptor mRNA to murine proximal tubular cells, followed by IGF-1 receptor synthesis and enhanced sensitivity to IGF-1, provided an explanation for the amplification of the regenerative action of the few MSCs localized to the kidney [80], and further supported the notion that exRNA is transferred via EVs in AKI [38]. The efficacy of MSC-derived EVs was also observed in other models of renal injury. Human umbilical MSC-derived EVs were also shown to activate the extracellular signal-regulated kinase (ERK) 1/2 pathway, which is involved in tubular cell proliferation and protection from cisplatin-induced apoptosis [81]. In the mouse model of remnant kidney, which is characterized by development of chronic renal disease, EVs released by MSCs have also been shown to prevent fibrosis [82]. We have previously reported a protective effect of MSC-derived EVs in cisplatin-induced lethal AKI [83] where EVs significantly improved survival of mice. The underlying explanation was that EVs induced upregulation of genes that antagonize apoptosis (Bcl-xL, Bcl2, and BIRC8) and downregulation of proapoptotic genes (Casp1, Casp8, and LTA) [83].

Phenotypic changes induced by MSC-EVs have also been shown to promote regeneration in other organs including the liver, lung, and heart. For example, in a model of 70 % hepatectomy, EVs promoted liver morphological and functional recovery through the transfer of specific subsets of mRNA, associated with the control of transcription, translation, proliferation, and apoptosis [39]. In addition, using human AGO2 as a reporter gene present in EVs showed that the human protein was translated from AGO2 mRNA which was incorporated into the liver of EV-treated rats. Other studies have demonstrated that MSC-derived EVs may stimulate liver regeneration by activation of the interleukin-6/STAT3 pathway [84] and reduce liver fibrosis [85] in CCl4-induced injury.

Furthermore, in an endotoxin-induced murine model of acute lung injury, the beneficial effect of treatment with MSC-derived EVs was attributed to the transfer of keratinocyte growth factor (KGF) mRNA to the injured alveolar epithelial cells [86]. In fact, EVs depleted of KGF mRNA by transfection of MSC with a specific siRNA were significantly less effective in reparation.

Timmers et al. [87] showed that administering MSC-conditioned medium after ischemia/reperfusion injury (IRI) in the heart reduced the infarct size in a murine model of myocardial infarction. Lai et al. [88] provided evidence that EVs present in MSC-conditioned medium were responsible for cardioprotection. Internalization into target cells at the infarct site was shown to be a requirement, because homogenized EVs were no longer cardioprotective [89]. Borges et al. demonstrated that transforming growth factor-β1 mRNA transported by EVs may activate both repair/regenerative responses and fibrosis by fibroblast activation [90].

Enrichment of miRNAs in MSC-EVs [19, 46, 48] suggests that these noncoding posttranscriptional modulators of gene expression are candidates for potential effectors of EVs. We investigated whether there was any modulation in miRNAs by MSC-EVs in renal tubular epithelial cells exposed to IRI induced by ATP depletion [91]. Changes in miRNA expression observed after injury were reverted by EV administration. EV-dependent modulation of miRNAs was partly dependent on miRNA transfer via EVs, and partly due to EV-triggered transcription. In particular, it was found that EVs transferred miRNAs and/or enhanced the expression of miRNAs which downregulated apoptosis and cell death.

In an in-vivo model of AKI, we found that MSC-EVs counteracted alterations in mRNA levels, detected by deep sequence analyses in injured kidneys [91]. This effect of EVs, which was associated with morphological and functional recovery, was dependent on EV miRNA content. In fact, miRNA-depleted EVs generated by Drosha knockdown in MSCs were devoid of healing properties [92], suggesting that the miRNA content of EVs is crucial for its biological activity. The role of miRNAs was confirmed in a murine model of renal IRI [93] and in a model of hind-limb ischemia [94] treated with EVs from wild-type or Dicer knockdown endothelial progenitor cells (EPCs) to impair the expression of miRNAs in EVs. In these models, only EVs from wild-type EPCs were renoprotective and improved neovascularization. The involvement of angiogenic miR-126 and miR-296 was suggested by experiments which demonstrated that silencing of these miRNAs abrogated EV activity [93].

Pulmonary hypertension has multiple disease associations and is a serious and eventually lethal condition. Pulmonary hypertension is characterized by vascular remodeling and right ventricular hypertrophy. Aliotta et al. [95] have shown that lung-derived or plasma-derived vesicles from mice with monocrotaline-induced pulmonary hypertension could induce pulmonary hypertension in normal mice. Whether this is due to a direct effect on vascular remodeling in the lung or due to an indirect effect through the marrow is the subject of ongoing investigations. The effect of marrow MSC-derived vesicles on monocrotaline-induced pulmonary hypertension is also under investigation. Human or murine marrow MSC-derived vesicles have also been shown to partially reverse radiation damage due to murine marrow cells in vitro and in vivo*.*

Nakamura et al. [96] recently provided evidence that MSC-derived exosomes enhance myogenesis and angiogenesis promoting muscle regeneration by a mechanism at least partially mediated by miR-494. EV-mediated delivery of miR-133b from MSCs to neurons and astrocytes has also been implicated in the induction of neurite outgrowth both in vitro and in vivo [97, 98]. EV transfer of miR-221 from MSCs to cardiomyocytes has been shown to confer cardioprotection by targeting p53-upregulated modulator of apoptosis (PUMA) [99].

Taken together, these studies suggest that EVs derived from stem/progenitor cells may stimulate tissue regeneration by modulation of gene transcription and induction of epigenetic changes in recipient cells [100].

Factors other than the delivery of exRNAs, however, are involved in the injury protection and regeneration induced by stem/progenitor cell-derived EVs. Studies carried out by the Quesenberry group have demonstrated that the phenotypic alterations observed in bone marrow cells were dependent on their cell cycle status and on the injury of the originator cells [73]. In particular, it was found that the expression of adhesion molecules, allowing EVs to enter bone marrow stem cells, depends on the cell cycle and on the treatment of the cell of origin.

In addition, a recent comprehensive study on the content of MSC-derived EVs has shown that, beside miRNAs, EVs carry more than 150 different proteins including growth factors, modulators of extracellular matrix, and metabolites such as lactic and glutamic acid. Moreover, EVs were shown to contain biologically active lipids such as sphingomyelin that may be involved in EV biological activities [101]. We found that EVs released from MSCs derived from adipose tissue are enriched in c-kit, stem cell factor, and metalloproteinases, which favor angiogenic activity [102]. Culture conditions modulated the composition of EVs and their biological activity. Whereas platelet-derived growth factor was shown to enhance the presence of proangiogenic factors [102], fibroblast growth factor upregulated the expression of the anti-angiogenic factors and decreased the level of proangiogenic factors and of neoangiogenesis [103]. MSC-derived EVs also carry Wnt4 protein that has been shown to induce β-catenin activation in endothelial cells and angiogenesis favoring cutaneous wound healing [104]. Moreover, EVs containing annexin A1 were shown to activate wound regeneration circuits able to repair chronic mucosal injury [105].

Taken together, these studies clearly indicate that stem/progenitor cell-derived EVs have regenerative potential. However, it is not easy to compare different studies to understand the molecular mechanism implicated because of the different techniques used for purification and quantitation of EVs, the different cell types, and the different culture conditions. In addition, vesicles from the same cell type are heterogeneous in nature and the molecular content and the biological activity vary depending on stimulation. Whereas the exogenous administration of EVs has been proved to be effective in vivo in different experimental settings, it is not easy to determine whether EVs have a relevant in-vivo physiological importance in cell differentiation and reprogramming. The amount of circulating vesicles mainly derived from platelets, and to a lesser extent from monocytes and endothelial cells, largely exceeds doses normally used in vitro. It is more difficult to define the locally released EVs in different tissues under physiological and pathological conditions. The presence in tissue of vesicles released from cardiac telocytes has been shown by electron microscopy and electron tomography [106]. Moreover, some studies have been conducted to try to understand the potential contribution of released EVs to MSC paracrine action. The amount of EVs released in vitro overnight from 75,000 MSCs and injected intravenously was shown to mimic the beneficial effect of the same amount of cells in a model of AKI [38]. Quantitation by NanoSight (Malvern Instruments Ltd., Malvern, UK) of EVs produced in vitro by MSCs cultured in serum-free basal medium indicated the production of approximately 2200 vesicles per single cell in 12 h [91]. It should be underlined that the timing of EV collection may give different results because most of the released vesicles are re-uptaken by the producing cells. The effective production under physiological conditions within tissue cannot be determined, but it is conceivable that production of EVs varies upon stimulation and that locally released EVs act primarily on adjacent cells and synergize with other environmental stimuli in determining the cell fate.

## Conclusions: translational potential of extracellular vesicles

The considerations presented in this review suggest that EVs may either modify neighboring cell function and phenotypes within a defined microenvironment or act on distant cells following transportation by biological fluids. By delivering bioactive lipids, proteins, and nucleic acids, EVs may transfer the imprinting of the originator cells to the recipient cells. In the context of stem cell biology, this mechanism may account for stem tissue-injured cell communication. The influence can also be bidirectional, because tissue-injured cells may induce gene expression and differentiation decisions in the stem cells. Conversely, stem cell-derived vesicles may reprogram injured cells by activating regenerative mechanisms. In particular, the transfer of transcriptional factors and translational regulators, such as noncoding RNAs, may induce epigenetic modifications into recipient cells, which could be exploited in regenerative medicine. Based on these factors, it is important to fully understand the mechanisms involved in EV biogenesis and in changes in EV composition, dependent on environmental stimuli, in order to design possible new therapeutic interventions.

## Note

This article is part of a thematic series on *Extracellular vesicles and regenerative medicine* edited by Jeffrey Karp, Kelvin Ng and Armand Keating. Other articles in this series can be found at http://stemcellres.com/series/EVRM.

## Abbreviations

Ago2: Argonaute 2
AKI: Acute kidney injury
EBV: Epstein–Barr virus
ERK: Extracellular signal-regulated kinase
EPC: Endothelial progenitor cell
EV: Extracellular vesicle
exRNA: Extracellular RNA
GFP: Green fluorescent protein
IGF-1: Insulin-like growth factor 1
IRI: Ischemia/reperfusion injury
KGF: Keratinocyte growth factor
lncRNA: Long noncoding RNA
miRNA: MicroRNA
MSC: Mesenchymal stromal cell
PUMA: p53-upregulated modulator of apoptosis

## References

[1] Sherer NM, Mothes W (2008). Cytonemes and tunnelling nanotubules in cell–cell communication and viral pathogenesis. Trends Cell Biol..

[2] Peinado H, Lavotshkin S, Lyden D (2011). The secreted factors responsible for pre-metastatic niche formation: old sayings and new thoughts. Semin Cancer Biol..

[3] Discher DE, Mooney DJ, Zandstra PW (2009). Growth factors, matrices, and forces combine and control stem cells. Science..

[4] Murry CE, Keller G (2008). Differentiation of embryonic stem cells to clinically relevant populations: lessons from embryonic development. Cell..

[5] Boyer LA, Mathur D, Jaenisch R (2006). Molecular control of pluripotency. Curr Opin Genet Dev..

[6] Buganim Y, Faddah DA, Jaenisch R (2013). Mechanisms and models of somatic cell reprogramming. Nat Rev Genet..

[7] Ratajczak J, Wysoczynski M, Hayek F, Janowska-Wieczorek A, Ratajczak MZ (2006). Membrane-derived microvesicles: important and underappreciated mediators of cell-to-cell communication. Leukemia..

[8] Cocucci E, Racchetti G, Meldolesi J (2008). Shedding microvesicles: artefacts no more. Trends Cell Biol..

[9] Colombo M, Raposo G, Théry C (2014). Biogenesis, secretion, and intercellular interactions of exosomes and other extracellular vesicles. Annu Rev Cell Dev Biol..

[10] Gould SJ, Raposo G. As we wait: coping with an imperfect nomenclature for extracellular vesicles. J Extracell Vesicles. 2013;2. doi:10.3402/jev.v2i0.20389. eCollection 2013. 10.3402/jev.v2i0.20389 PMC3760635 24009890

[11] Lee TH, D'Asti E, Magnus N, Al-Nedawi K, Meehan B, Rak J (2011). Microvesicles as mediators of intercellular communication in cancer—the emerging science of cellular “debris”. Semin Immunopathol..

[12] Bobrie A, Colombo M, Raposo G, Théry C (2011). Exosome secretion: molecular mechanisms and roles in immune responses. Traffic..

[13] Raposo G, Nijman HW, Stoorvogel W, Liejendekker R, Harding CV, Melief CJ (1996). B lymphocytes secrete antigen-presenting vesicles. J Exp Med..

[14] Zitvogel L, Regnault A, Lozier A, Wolfers J, Flament C, Tenza D (1998). Eradication of established murine tumors using a novel cell-free vaccine: dendritic cell-derived exosomes. Nat Med..

[15] Quah BJ, Barlow VP, McPhun V, Matthaei KI, Hulett MD, Parish CR (2008). Bystander B cells rapidly acquire antigen receptors from activated B cells by membrane transfer. Proc Natl Acad Sci U S A..

[16] Janowska-Wieczorek A, Majka M, Kijowski J, Baj-Krzyworzeka M, Reca R, Turner AR (2001). Platelet-derived microparticles bind to hematopoietic progenitor cells and enhance their engraftment. Blood..

[17] Barry OP, Praticò D, Savani RC, FitzGerald GA (1998). Modulation of monocyte–endothelial cell interactions by platelet microparticles. J Clin Invest..

[18] Kim JW, Wieckowski E, Taylor DD, Reichert TE, Watkins S, Whiteside TL (2005). Fas ligand-positive membranous vesicles isolated from sera of patients with oral cancer induce apoptosis of activated T lymphocytes. Clin Cancer Res..

[19] Castellana D, Kunzelmann C, Freyssinet JM (2009). Pathophysiologic significance of procoagulant microvesicles in cancer disease and progression. Hamostaseologie..

[20] Kim CW, Lee HM, Lee TH, Kang C, Kleinman HK, Gho YS (2002). Extracellular membrane vesicles from tumor cells promote angiogenesis via sphingomyelin. Cancer Res..

[21] Castellana D, Zobairi F, Martinez MC, Panaro MA, Mitolo V, Freyssinet JM (2009). Membrane microvesicles as actors in the establishment of a favorable prostatic tumoral niche: a role for activated fibroblasts and CX3CL1–CX3CR1 axis. Cancer Res..

[22] Antonyak MA, Li B, Boroughs LK, Johnson JL, Druso JE, Bryant KL (2011). Cancer cell-derived microvesicles induce transformation by transferring tissue transglutaminase and fibronectin to recipient cells. Proc Natl Acad Sci U S A..

[23] Hood JL, San RS, Wickline SA (2011). Exosomes released by melanoma cells prepare sentinel lymph nodes for tumor metastasis. Cancer Res..

[24] Jung T, Castellana D, Klingbeil P, Cuesta Hernández I, Vitacolonna M, Orlicky DJ (2009). CD44v6 dependence of premetastatic niche preparation by exosomes. Neoplasia..

[25] Grange C, Tapparo M, Collino F, Vitillo L, Damasco C, Deregibus MC (2011). Microvesicles released from human renal cancer stem cells stimulate angiogenesis and formation of lung premetastatic niche. Cancer Res..

[26] Bhat R, Bissell MJ (2014). Of plasticity and specificity: dialectics of the microenvironment and macroenvironment and the organ phenotype. Wiley Interdiscip Rev Dev Biol..

[27] Quesenberry PJ, Aliotta JM (2010). Cellular phenotype switching and microvesicles. Adv Drug Deliv Rev..

[28] Chen X, Liang H, Zhang J, Zen K, Zhang CY (2012). Secreted microRNAs: a new form of intercellular communication. Trends Cell Biol..

[29] Gallo A, Tandon M, Alevizos I, Illei GG (2012). The majority of microRNAs detectable in serum and saliva is concentrated in exosomes. PLoS One..

[30] Wang K, Zhang S, Weber J, Baxter D, Galas DJ (2010). Export of microRNAs and microRNA-protective protein by mammalian cells. Nucleic Acids Res..

[31] Arroyo JD, Chevillet JR, Kroh EM, Ruf IK, Pritchard CC, Gibson DF (2011). Argonaute2 complexes carry a population of circulating microRNAs independent of vesicles in human plasma. Proc Natl Acad Sci U S A..

[32] Vickers KC, Palmisano BT, Shoucri BM, Shamburek RD, Remaley AT (2011). MicroRNAs are transported in plasma and delivered to recipient cells by high-density lipoproteins. Nat Cell Biol..

[33] Guallar D, Wang J (2014). RNA-binding proteins in pluripotency, differentiation, and reprogramming. Front Biol (Beijing).

[34] Li Z, Dang J, Chang KY, Rana TM (2014). MicroRNA-mediated regulation of extracellular matrix formation modulates somatic cell reprogramming. RNA..

[35] Ratajczak J, Miekus K, Kucia M, Zhang J, Reca R, Dvorak P (2006). Embryonic stem cell-derived microvesicles reprogram hematopoietic progenitors: evidence for horizontal transfer of mRNA and protein delivery. Leukemia..

[36] Deregibus MC, Cantaluppi V, Calogero R, Lo Iacono M, Tetta C, Biancone L (2007). Endothelial progenitor cell derived microvesicles activate an angiogenic program in endothelial cells by a horizontal transfer of mRNA. Blood..

[37] Valadi H, Ekstrom K, Bossios A, Sjostrand M, Lee JJ, Lotvall JO (2007). Exosome-mediated transfer of mRNAs and microRNAs is a novel mechanism of genetic exchange between cells. Nat Cell Biol..

[38] Bruno S, Grange C, Deregibus MC, Calogero RA, Saviozzi S, Collino F (2009). Mesenchymal stem cell-derived microvesicles protect against acute tubular injury. J Am Soc Nephrol..

[39] Herrera MB, Fonsato V, Gatti S, Deregibus MC, Sordi A, Cantarella D (2010). Human liver stem cell-derived microvesicles accelerate hepatic regeneration in hepatectomized rats. J Cell Mol Med..

[40] Aliotta JM, Pereira M, Johnson KW, de Paz N, Dooner MS, Puente N (2010). Microvesicle entry into marrow cells mediates tissue-specific changes in mRNA by direct delivery of mRNA and induction of transcription. Exp Hematol..

[41] Ridder K, Keller S, Dams M, Rupp AK, Schlaudraff J, Turco DD (2014). Extracellular vesicle-mediated transfer of genetic information between the hematopoietic system and the brain in response to inflammation. PLoS Biol..

[42] Katsman D, Stackpole EJ, Domin DR, Farber DB (2012). Embryonic stem cell-derived microvesicles induce gene expression changes in Müller cells of the retina. PLoS One.

[43] Irmak MK, Oztas Y, Oztas E (2012). Integration of maternal genome into the neonate genome through breast milk mRNA transcripts and reverse transcriptase. Theor Biol Med Model..

[44] Batagov AO, Kurochkin IV (2013). Exosomes secreted by human cells transport largely mRNA fragments that are enriched in the 3′-untranslated regions. Biol Direct..

[45] Whitehead J, Pandey GK, Kanduri C (2009). Regulation of the mammalian epigenome by long noncoding RNAs. Biochim Biophys Acta..

[46] Yuan A, Farber EL, Rapoport AL, Tejada D, Deniskin R, Akhmedov NB (2009). Transfer of microRNAs by embryonic stem cell microvesicles. PLoS One..

[47] Chen TS, Lai RC, Lee MM, Choo AB, Lee CN, Lim SK (2010). Mesenchymal stem cell secretes microparticles enriched in pre-microRNAs. Nucleic Acids Res..

[48] Collino F, Deregibus MC, Bruno S, Sterpone L, Aghemo G, Viltono L (2010). Microvesicles derived from adult human bone marrow and tissue specific mesenchymal stem cells shuttle selected pattern of miRNAs. PLoS One..

[49] Gibbings DJ, Ciaudo C, Erhardt M, Voinnet O (2009). Multivesicular bodies associate with components of miRNA effector complexes and modulate miRNA activity. Nat Cell Biol..

[50] Laffont B, Corduan A, Plé H, Duchez AC, Cloutier N, Boilard E (2013). Activated platelets can deliver mRNA regulatory Ago2–microRNA complexes to endothelial cells via microparticles. Blood..

[51] Li L, Zhu D, Huang L, Zhang J, Bian Z, Chen X (2012). Argonaute 2 complexes selectively protect the circulating microRNAs in cell-secreted microvesicles. PLoS One..

[52] Lv Z, Wei Y, Wang D, Zhang CY, Zen K, Li L (2014). Argonaute 2 in cell-secreted microvesicles guides the function of secreted miRNAs in recipient cells. PLoS One..

[53] Guduric-Fuchs J, O'Connor A, Camp B, O'Neill CL, Medina RJ, Simpson DA (2012). Selective extracellular vesicle-mediated export of an overlapping set of microRNAs from multiple cell types. BMC Genomics..

[54] Vickers KC, Remaley AT (2012). Lipid-based carriers of microRNAs and intercellular communication. Curr Opin Lipidol..

[55] Kosaka N, Iguchi H, Yoshioka Y, Takeshita F, Matsuki Y, Ochiya T (2010). Secretory mechanisms and intercellular transfer of microRNAs in living cells. J Biol Chem..

[56] Kosaka N, Iguchi H, Yoshioka Y, Hagiwara K, Takeshita F, Ochiya T (2012). Competitive interactions of cancer cells and normal cells via secretory microRNAs. J Biol Chem..

[57] Pegtel DM, van de Garde MD, Middeldorp JM (1809). Viral miRNAs exploiting the endosomal–exosomal pathway for intercellular cross-talk and immune evasion. Biochim Biophys Acta..

[58] Zhang Y, Liu D, Chen X, Li J, Li L, Bian Z (2010). Secreted monocytic miR-150 enhances targeted endothelial cell migration. Mol Cell..

[59] Mittelbrunn M, Gutiérrez-Vázquez C, Villarroya-Beltri C, González S, Sánchez-Cabo F, González MÁ (2011). Unidirectional transfer of microRNA-loaded exosomes from T cells to antigen-presenting cells. Nat Commun..

[60] Montecalvo A, Larregina AT, Shufesky WJ, Stolz DB, Sullivan ML, Karlsson JM (2012). Mechanism of transfer of functional microRNAs between mouse dendritic cells via exosomes. Blood..

[61] Waldenström A, Gennebäck N, Hellman U, Ronquist G (2012). Cardiomyocyte microvesicles contain DNA/RNA and convey biological messages to target cells. PLoS One..

[62] Guescini M, Genedani S, Stocchi V, Agnati LF (2010). Astrocytes and glioblastoma cells release exosomes carrying mtDNA. J Neural Transm..

[63] Balaj L, Lessard R, Dai L, Cho YJ, Pomeroy SL, Breaqkfield XO (2011). Tumour microvesicles contain retrotransposon elements and amplified oncogene sequences. Nat Commun..

[64] Lee TH, Chennakrishnaiah S, Audemard E, Montermini L, Meehan B, Rak J (2014). Oncogenic ras-driven cancer cell vesiculation leads to emission of double-stranded DNA capable of interacting with target cells. Biochem Biophys Res Commun..

[65] Al-Nedawi K, Meehan B, Micallef J, Lhotak V, May L, Guha A (2008). Intercellular transfer of the oncogenic receptor EGFRvIII by microvesicles derived from tumour cells. Nat Cell Biol..

[66] Quesenberry PJ, Dooner G, Dooner M, Abedi M (2005). Developmental biology: Ignoratio elenchi: red herrings in stem cell research. Science..

[67] Abedi M, Greer DA, Colvin GA, Demers DA, Dooner MS, Harpel JA (2004). Robust conversion of marrow cells to skeletal muscle with formation of marrow-derived muscle cell colonies: a multifactorial process. Exp Hematol..

[68] Dooner M, Cerny J, Colvin G, Demers D, Pimentel J, Greer D (2004). Homing and conversion of murine hematopoietic stem cells to lung. Blood Cells Mol Dis..

[69] Badiavas EV, Abedi M, Butmarc J, Falanga V, Quesenberry P (2003). Participation of bone marrow derived cells in cutaneous wound healing. J Cell Physiol..

[70] Aliotta JM, Keaney P, Passero M, Dooner MS, Pimentel J, Greer D (2006). Bone marrow production of lung cells: the impact of G-CSF, cardiotoxin, graded doses of irradiation, and subpopulation phenotype. Exp Hematol..

[71] Aliotta JM, Sanchez-Guijo FM, Dooner GJ, Johnson KW, Dooner MS, Greer KA (2007). Alteration of marrow cell gene expression, protein production, and engraftment into lung by lung-derived microvesicles: a novel mechanism for phenotype modulation. Stem Cells..

[72] Aliotta JM, Pereira M, Li M, Amaral A, Sorokina A, Dooner MS, et al. Stable cell fate changes in marrow cells induced by lung-derived microvesicles. J Extracell Vesicles. 2012;1. doi:10.3402/jev.v1i0.18163. eCollection 2012. 10.3402/jev.v1i0.18163 PMC3760634 24009878

[73] Aliotta JM, Lee D, Puente N, Faradyan S, Sears EH, Amaral A (2012). Progenitor/stem cell fate determination: interactive dynamics of cell cycle and microvesicles. Stem Cells Dev..

[74] Quesenberry PJ, Dooner MS, Aliotta JM (2010). Stem cell plasticity revisited: the continuum marrow model and phenotypic changes mediated by microvesicles. Exp Hematol..

[75] Poulsom R, Forbes SJ, Hodivala-Dilke K, Ryan E, Wyles S, Navaratnarasah S (2001). Bone marrow contributes to renal parenchymal turnover and regeneration. J Pathol..

[76] Humphreys BD, Valerius MT, Kobayashi A, Mugford JW, Soeung S, Duffield JS (2008). Intrinsic epithelial cells repair the kidney after injury. Cell Stem Cell..

[77] Ishibe S, Cantley LG (2008). Epithelial–mesenchymal–epithelial cycling in kidney repair. Curr Opin Nephrol Hypertens..

[78] Bi B, Schmitt R, Israilova M, Nishio H, Cantley LG (2007). Stromal cells protect against acute tubular injury via an endocrine effect. J Am Soc Nephrol..

[79] Otto WR, Wright NA (2011). Mesenchymal stem cells: from experiment to clinic. Fibrogenesis Tissue Repair..

[80] Tomasoni S, Longaretti L, Rota C, Morigi M, Conti S, Gotti E (2013). Transfer of growth factor receptor mRNA via exosomes unravels the regenerative effect of mesenchymal stem cells. Stem Cells Dev..

[81] Zhou Y, Xu H, Xu W, Wang B, Wu H, Tao Y (2013). Exosomes released by human umbilical cord mesenchymal stem cells protect against cisplatin-induced renal oxidative stress and apoptosis in vivo and in vitro. Stem Cell Res Ther..

[82] He J, Wang Y, Sun S, Yu M, Wang C, Pei X (2012). Bone marrow stem cells-derived microvesicles protect against renal injury in the mouse remnant kidney model. Nephrology (Carlton).

[83] Bruno S, Grange C, Collino F, Deregibus MC, Cantaluppi V, Biancone L (2012). Microvesicles derived from mesenchymal stem cells enhance survival in a lethal model of acute kidney injury. PLoS One..

[84] Tan CY, Lai RC, Wong W, Dan YY, Lim SK, Ho HK (2014). Mesenchymal stem cell-derived exosomes promote hepatic regeneration in drug-induced liver injury models. Stem Cell Res Ther..

[85] Li T, Yan Y, Wang B, Qian H, Zhang X, Shen L (2013). Exosomes derived from human umbilical cord mesenchymal stem cells alleviate liver fibrosis. Stem Cells Dev..

[86] Zhu YG, Feng XM, Abbott J, Fang XH, Hao Q, Monsel A (2014). Human mesenchymal stem cell microvesicles for treatment of Escherichia coli endotoxin-induced acute lung injury in mice. Stem Cells..

[87] Timmers L, Lim SK, Arslan F, Armstrong JS, Hoefer IE, Doevendans PA (2007). Reduction of myocardial infarct size by human mesenchymal stem cell conditioned medium. Stem Cell Res..

[88] Lai RC, Chen TS, Lim SK (2011). Mesenchymal stem cell exosome: a novel stem cell-based therapy for cardiovascular disease. Regen Med..

[89] Arslan F, Lai RC, Smeets MB, Akeroyd L, Choo A, Aguor EN (2013). Mesenchymal stem cell-derived exosomes increase ATP levels, decrease oxidative stress and activate PI3K/Akt pathway to enhance myocardial viability and prevent adverse remodeling after myocardial ischemia/reperfusion injury. Stem Cell Res..

[90] Borges FT, Melo SA, Özdemir BC, Kato N, Revuelta I, Miller CA (2013). TGF-β1-containing exosomes from injured epithelial cells activate fibroblasts to initiate tissue regenerative responses and fibrosis. J Am Soc Nephrol..

[91] Lindoso RS, Collino F, Bruno S, Araujo DS, Sant'Anna JF, Tetta C (2014). Extracellular vesicles released from mesenchymal stromal cells modulate miRNA in renal tubular cells and inhibit ATP depletion injury. Stem Cells Dev..

[92] Collino F, Bruno S, Incarnato D, Dettori D, Neri F, Provero P, Pomatto M, Oliviero S, Tetta C, Quesenberry P, Camussi G. Acute kidney injury recovery induced by extracellular vesicles carrying miRNA. J Am Soc Nephrol. 2015. [Epub ahead of print]. 10.1681/ASN.2014070710 PMC4587694 25901032

[93] Cantaluppi V, Gatti S, Medica D, Figliolini F, Bruno S, Deregibus MC (2012). Microvesicles derived from endothelial progenitor cells protect the kidney from ischemia-reperfusion injury by microRNA-dependent reprogramming of resident renal cells. Kidney Int..

[94] Ranghino A, Cantaluppi V, Grange C, Vitillo L, Fop F, Biancone L (2012). Endothelial progenitor cell-derived microvesicles improve neovascularization in a murine model of hindlimb ischemia. Int J Immunopathol Pharmacol..

[95] Aliotta JM, Pereira M, Amaral A, Sorokina A, Igbinoba Z, Hasslinger A (2013). Induction of pulmonary hypertensive changes by extracellular vesicles from monocrotaline-treated mice. Cardiovasc Res..

[96] Nakamura Y, Miyaki S, Ishitobi H, Matsuyama S, Nakasa T, Kamei N (2015). Mesenchymal-stem-cell-derived exosomes accelerate skeletal muscle regeneration. FEBS Lett..

[97] Xin H, Li Y, Buller B, Katakowski M, Zhang Y, Wang X (2012). Exosome-mediated transfer of miR-133b from multipotent mesenchymal stromal cells to neural cells contributes to neurite outgrowth. Stem Cells..

[98] Xin H, Li Y, Liu Z, Wang X, Shang X, Cui Y (2013). Mir-133b promotes neural plasticity and functional recovery after treatment of stroke with multipotent mesenchymal stromal cells in rats via transfer of exosome enriched extracellular particles. Stem Cells..

[99] Yu B, Gong M, Wang Y, Millard RW, Pasha Z, Yang Y (2013). Cardiomyocyte protection by GATA-4 gene engineered mesenchymal stem cells is partially mediated by translocation of miR-221 in microvesicles. PLoS One..

[100] Quesenberry PJ, Goldberg LR, Aliotta JM, Dooner MS, Pereira MG, Wen S (2014). Cellular phenotype and extracellular vesicles: basic and clinical considerations. Stem Cells Dev..

[101] Vallabhaneni KC, Penfornis P, Dhule S, Guillonneau F, Adams KV, Mo YY (2015). Extracellular vesicles from bone marrow mesenchymal stem/stromal cells transport tumor regulatory microRNA, proteins, and metabolites. Oncotarget..

[102] Lopatina T, Bruno S, Tetta C, Kalinina N, Porta M, Camussi G (2014). Platelet-derived growth factor regulates the secretion of extracellular vesicles by adipose mesenchymal stem cells and enhances their angiogenic potential. Cell Commun Signal..

[103] Lopatina T, Mazzeo A, Bruno S, Tetta C, Kalinina N, Romagnoli R (2014). The angiogenic potential of adipose mesenchymal stem cell-derived extracellular vesicles is modulated by basic fibroblast growth factor. J Stem Cell Res Ther..

[104] Zhang B, Wu X, Zhang X, Sun Y, Yan Y, Shi H (2015). Human umbilical cord mesenchymal stem cell exosomes enhance angiogenesis through the Wnt4/β-catenin pathway. Stem Cells Transl Med..

[105] Leoni G, Neumann PA, Kamaly N, Quiros M, Nishio H, Jones HR (2015). Annexin A1-containing extracellular vesicles and polymeric nanoparticles promote epithelial wound repair. J Clin Invest..

[106] Fertig ET, Gherghiceanu M, Popescu LM (2014). Extracellular vesicles release by cardiac telocytes: electron microscopy and electron tomography. J Cell Mol Med..

